# Insights from modeling studies on how climate change affects invasive alien species geography

**DOI:** 10.1002/ece3.4098

**Published:** 2018-05-04

**Authors:** Celine Bellard, Jonathan M. Jeschke, Boris Leroy, Georgina M. Mace

**Affiliations:** ^1^ Department of Genetics, Evolution and Environment Centre for Biodiversity and Environment Research London UK; ^2^ Unité Biologie des organismes et écosystèmes aquatiques (BOREA UMR 7208) Muséum national d'Histoire naturelle CNRS, IRD Sorbonne Universités, Université Pierre et Marie Curie, Université de Caen Normandie, Université des Antilles Paris France; ^3^ Leibniz‐Institute of Freshwater Ecology and Inland Fisheries (IGB) Berlin Germany; ^4^ Department of Biology, Chemistry, Pharmacy Institute of Biology Freie Universität Berlin Berlin Germany; ^5^ Berlin‐Brandenburg Institute of Advanced Biodiversity Research (BBIB) Berlin Germany

**Keywords:** biological invasions, climate change, future, range size, shifts, species distribution models

## Abstract

Climate change and biological invasions are threatening biodiversity and ecosystem services worldwide. It has now been widely acknowledged that climate change will affect biological invasions. A large number of studies have investigated predicted shifts and other changes in the geographic ranges of invasive alien species related to climate change using modeling approaches. Yet these studies have provided contradictory evidence, and no consensus has been reached. We conducted a systematic review of 423 modeling case studies included in 71 publications that have examined the predicted effects of climate change on those species. We differentiate the approaches used in these studies and synthesize their main results. Our results reaffirm the major role of climate change as a driver of invasive alien species distribution in the future. We found biases in the literature both regarding the taxa, toward plants and invertebrates, and the areas of the planet investigated. Despite these biases, we found for the plants and vertebrates studied that climate change will more frequently contribute to a decrease in species range size than an increase in the overall area occupied. This is largely due to oceans preventing terrestrial invaders from spreading poleward. In contrast, we found that the ranges of invertebrates and pathogens studied are more likely to increase following climate change. An important caveat to these findings is that researchers have rarely considered the effects of climate change on transport, introduction success, or the resulting impacts. We recommend closing these research gaps, and propose additional avenues for future investigations, as well as opportunities and challenges for managing invasions under climate change.

## 1 INTRODUCTION

Biological invasions are an important driver of biodiversity loss. During the last century, they have been associated with nearly 60% of species extinctions (Bellard, Cassey, & Blackburn, 2016). They also jeopardize ecosystem services and challenge human health and economic growth (Simberloff et al., 2013). Because of increasing trade and habitat disturbance, the pressure caused by biological invasions is likely to continue or even increase (Hulme, 2009).

Climate change is also expected to alter invasion processes. For instance, a higher frequency of extreme weather and the consequent opening of shipping lanes in the Arctic (Miller & Ruiz, 2014) may facilitate transport of alien species, sometimes with dramatic impacts on ecosystems and species of conservation concern. Climate change is additionally expected to alter the geographic distribution of invasive alien species (IAS) (Hellmann, Bierwagen, Dukes, & Byers, 2008). Because IAS species are typically generalists with broad climatic tolerances, they are generally considered likely to cope with climate change which will enable them to expand into new areas (Walther et al., 2009). Although there is limited evidence for current effects of climate change on IAS distribution, it is often suggested that climate change is one of the main drivers of future invasions (Bellard et al., 2013).

There is thus an urgent need to understand the relationship between climate change and IAS distributions, especially those that may have a major impact on biodiversity. Some authors have tackled this issue through opinion or review papers based on key examples (Hellmann et al., 2008; Walther et al., 2009) or used models to project the likely effects of climate change on the distribution of certain IAS. However, such studies are restricted to a single species or taxa. Some have conducted that climate change increases the area occupied by IAS (Barbet‐Massin et al., 2013; Gilioli, Pasquali, Parisi, & Winter, 2014; Kriticos, Sutherst, Brown, Adkins, & Maywald, 2003), while others show that climate change limits IAS distributions (Bellard et al., 2013; Bradley, Oppenheimer, & Wilcove, 2009; Xu, Feng, Yang, Zheng, & Zhang, 2013). Moreover, the multiplicity of species studied, the variable approaches used, and the resulting variability in projections make it difficult to get a clear picture of the future effects of climate change on invasions. Large‐scale analyses of their geographic responses to global climate change are rare (Bellard et al., 2013; Peterson, Stewart, Mohamed, & Arau, 2008). Thus, the key question about the likely effects (direction and strength) of climate change on IAS distributions remains unanswered.

To address this question, we undertook a review of modeling studies that explore how future climate change is predicted to affect some IAS. Specifically, we analyze data on predicted range shifts to examine the change in the potential range size of species due to climate change. We also describe the major biases and caveats of current approaches. We synthesize these studies’ results focusing on how climate and taxonomic group are associated with the projected change in IAS distribution for these studies. We also look for gaps in existing studies in order to guide future research to improve our understanding of climate change effects on biological invasions.

## 2 MATERIALS AND METHODS

Developing our analyses involves three steps: (1) literature searches using search terms related to biological invasions and climate change, screening titles and abstracts to remove studies that address unrelated topics, and selecting studies that report the effects of at least one climate component on the studied species; (2) data collection: extracting and collecting information on the predicted size of the species range size change and, when available, the direction of range shifts; modeling approaches, species group studied, identity of the species, and variables projected; and (3) data analysis. Details of each of these steps are provided in the following sections.

### 2.1 Literature search

On 23 February 2016, we searched the ISI Web of Science database for articles that examine the impact of climate change on biological invasions through modeling approaches using a combination of search terms for biological invasions and climate change. We used a range of terms related to IAS (taxonomic and invasion terms) and climate variables. The specific search term was as follows: ((invasi*) OR (invader) OR (non‐native) OR (exotic) OR (alien) OR (non‐indigenous) OR (introduced) OR (naturalised species) OR (naturalized species) OR (biological invasion*)) AND ((plant) OR (invertebrate) OR (vertebrate) OR (alga*) OR (bacteri*) OR (virus) OR (microorganism) OR (fung*)) AND ((climate change) OR (global warming) OR (temperature) OR (precipitation) OR (extreme event*) OR (carbon dioxide) OR (CO2)). We then refined our search to select only articles and proceedings papers that are related to biology. This search resulted in 6,911 articles.

After a careful screening of titles and abstracts, we removed those articles that addressed unrelated topics. Of the 155 remaining studies, we selected papers that met all the following criteria: They evaluated the impact of at least one climate component (temperature, precipitation, or extreme events) on IAS range size or area distribution and they predicted future effects of climate change (≥year 2020). This resulted in 71 studies (see Table S1 for detailed references). Given the many ways that such studies are reported, any set of search terms will only be able to identify a sample of studies and so this dataset should not be considered exhaustive (see discussion section for details).

### 2.2 Data collection

For these 71 studies, we collected the following information: (1) methodological approaches based on distribution data only like species distribution models (called distribution models), on species process and/or dynamic population models based on growth rate for example (called process models) or approaches that combined both distribution and process like Climex (called here combined models); (2) biological level studied (i.e., individuals, population, species, community); (3) variables recorded (climate including temperature and precipitation, land use, or others); (4) closest date of predicted range; (5) species identity; (6) geographical scale of the study (i.e., world, large region—area >1 million km^2^, small region—between 1 million km^2^ and 50,000 km^2^; local—<50,000 km^2^); (7) spatial resolution; and (8) response variables examined for the effects of climate change (percentage of predicted change in species range size and/or change in suitability value, and predicted shift of distribution, number of generations, growth, or abundance).

We extracted the values estimated for these response variables when they were provided by the studies or contacted the author to get for this information. One commonly used measure is the species range change (SRC) value which is the difference between the predicted species range size in the future following climate change and the predicted species range size for the current period (under current climatic conditions). In most cases, only one SRC value was available in the study (e.g., for one IPCC scenario or averaged across multiple scenarios). We collected the percentage or the value of the SRC for each case study and also classified the SRCs into three qualitative categories: increase (SRC > 0.5%), decrease (SRC ≤ 0.5%), or stable (SRC between −0.5% and 0.5%). Note that in spite of their critical importance, both the coordinate reference system and the correction for pixel surface are hardly ever indicated in studies. This is critical because the count of raw pixels without surface correction may result in inflated estimates of range increase at high latitudes, whereas estimates of range decrease at low latitudes might be too low. In addition, range size decreases can only have values between 0 and −100%, while potential increases in range have no upper limit, and therefore, potential increases can outweigh potential decreases.

For each study, we noted the general habitat of the organisms (i.e., terrestrial, freshwater, or marine) and the higher taxa to which they belong. The studies were mostly at a large spatial scale, but country assessments were biased toward upper‐middle and high‐income countries such as Australia, South Africa, United States, and European nations, which confirms previous bias observed in studies of biological invasions (Bellard & Jeschke, 2016). If any of these data could not be retrieved directly from the publication, we contacted the authors (they are acknowledged in the Acknowledgements). In addition, if multiple species were examined separately within the same article, they were treated as separate case studies, but we kept a record of the original study information. This procedure resulted in 423 case studies.

### 2.3 Data analyses

Because most of the data extracted reported species range change, we mainly focus our analyses on this metric. In particular, we compared binary predictions of SRC predicted to increase or decrease for each taxonomic group and scale of the study. We also calculated the median of SRC predicted (resulting from climate change) for each taxonomic group separately considering the scale of the study. We show boxplots of SRC representing the median, and upper and lower “hinges” corresponding to the first and third quartiles of SRC within taxa. If all the data were easily available, we would have conducted a formal meta‐analysis to establish confidence limits around the average effect size of SRC and to test for consistency or lack of agreements of SRC predicted across taxon and region. However, this type of analyses would require information on mean SRC, sample size, and standard error (Koricheva & Gurevitch, 2014) for each emission scenario and time period. Such data were not available in the published studies.

All statistical analyses were performed using R version 3.2.4 (R Team, 2016) and dplyr version 0.7.4 (Wickham & Francois, 2015) and ggplot2 packages (Wickham, 2009).

## 3 RESULTS

### 3.1 Main approaches and biases in assessing the future of species distribution

**Figure 1:**
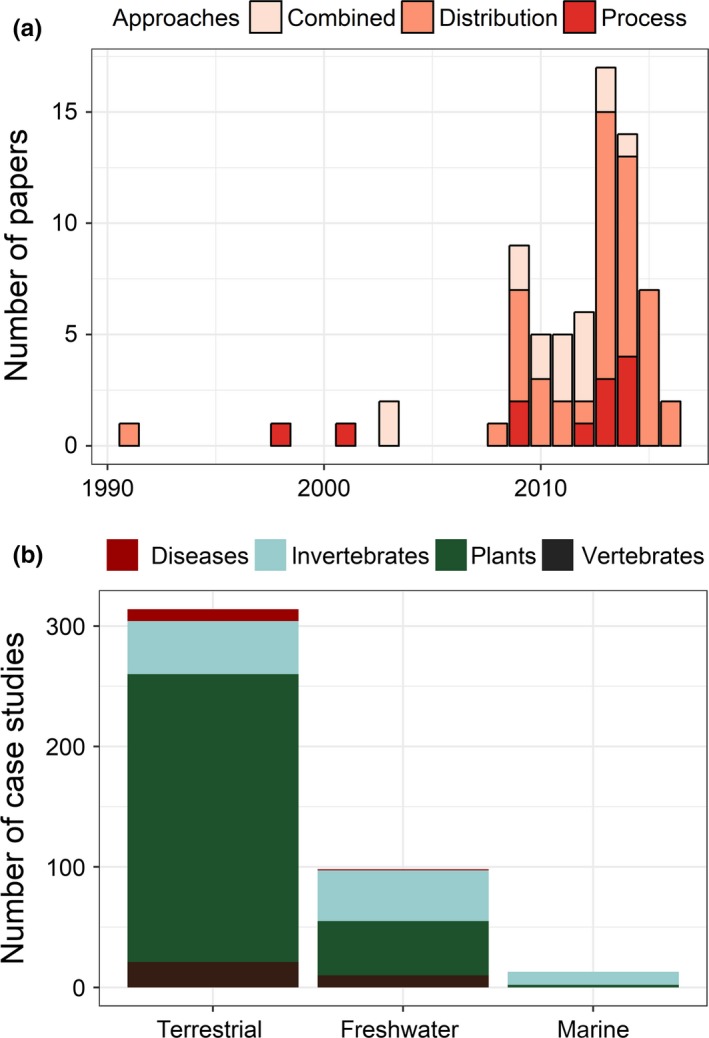
(a) Number of papers based on the three approaches from 1991 to February 2016. Specific colors indicate approaches used in each study (see text); (b) number of case studies per type of ecosystem and groups (that are identified by different colors)

In total, we found 71 modeling studies that investigated the future effects of climate change on IAS distributions. The number of these studies has increased over the last 10 years (Figure 1a). Most studies rely on species distribution models only (using MaxEnt or ensemble modeling through the BioMod platform, *n* = 43) or species distribution models combined with key processes like Climex, *n* = 16 (see Box 1 for modeling specificities).

Box 1 **We found that the three most frequently applied modeling approaches were MaxEnt, Climex, and ensemble modeling through the BioMod platform. These three approaches are outlined in the following paragraphs.**

**Correlative approaches**

- MaxEnt is a correlative approach that uses presence of background (i.e., species occurrences and environmental data such as climate), but not species absence data to estimate the association between species and environmental data based on the principle of *max*imum *ent*ropy (Phillips et al., 2006). The parameters in MaxEnt have no a priori defined ecological meaning and processes are implicit, which is in contrast to semi‐process‐based models such as Climex with explicit assumptions about mechanisms (Dormann et al., 2012). MaxEnt relies on different types of relationships (linear, quadratic, product, threshold) to link species data to environmental predictors (Phillips et al., 2006). This approach is known to perform relatively well, especially for small sample sizes (Elith et al., 2006), and can be applied to a wide range of species, including those with limited data. However, as for all correlative approaches, MaxEnt has potentially unrealistic extrapolation capacity (Dormann et al., 2012), which may be a weakness in estimating current and future potential invasive ranges.

**Process‐based approaches**

- Climex is a simplified dynamic model to simulate the mechanisms affecting geographic distributions at short time spans (days or weeks). Climex is based on species known geographic distributions, abundances, phenology, and climatic data (temperature, moisture, and light) and can be tuned on the basis of species known ecophysiology. This allows determination of the relative abundance and annual growth during the favorable season (see Kriticos et al., 2015, for a full description). The assumptions underlying Climex are biologically more realistic and have more potential for extrapolation in novel environments (e.g., future climates or uninvaded areas) and for modeling complex disease‐host range distributions than correlative distribution models. Climex includes growth indices, stress indices, and constraints to persistence that cover the major mechanisms by which terrestrial species respond to their environments. Yet, this approach requires more knowledge about the species being modeled, such as abundance data or ecophysiological knowledge, which may limit its applicability for species with no such data.

**Ensemble approaches**

- The ensemble modeling approach consists of producing multiple realizations of predictions (usually by combining multiple model classes with multiple sets of initial conditions, parameterizations, and scenarios), from which a consensus can be derived (e.g., average trend), and, most importantly, uncertainties can be quantified (Araújo & New, 2007). The rationale behind ensemble modeling is that identifying the best model in a given situation (e.g., current data) gives no certainty that this model will adequately represent new observations (e.g., future projections), which is specifically the case for extrapolations to novel environments. Ensemble modeling allows accounting for spatially explicit uncertainties in extrapolations by applying methods from information gap decision theory (Kujala, Burgman, & Moilanen, 2013) to guide management decisions. Yet, ensemble modeling based on correlative approaches is subject to a number of limitations that have been extensively discussed and include difficulties due to biotic interactions (Wisz et al., 2013) or dispersal abilities (Engler et al., 2009).

The majority of case studies focused on the establishment and spread phases of biological invasions only (cf. Blackburn et al., 2011), while the transport and introduction phases are often neglected even in areas where the species is not present yet. Only a few papers focused on other metrics for invasion risk, for example, changes in abundance (*N* = 5), growth (*N* = 1), or changes in the number of generations (*N* = 3) in areas.

Moreover, the majority (>310) of case studies explored the effect of climate change on species distributions in terrestrial ecosystems and they focused most on plants (*N* > 285), followed by invertebrates, vertebrates, and least commonly disease (Figure 1b). We found that few species had been studied more than once (the exception is *Lantana camara* that has been studied six times) and so there were few opportunities to compare and interpret results between different techniques and approaches.

### 3.2 Main trends regarding climate change and species distribution

**Figure 2:**
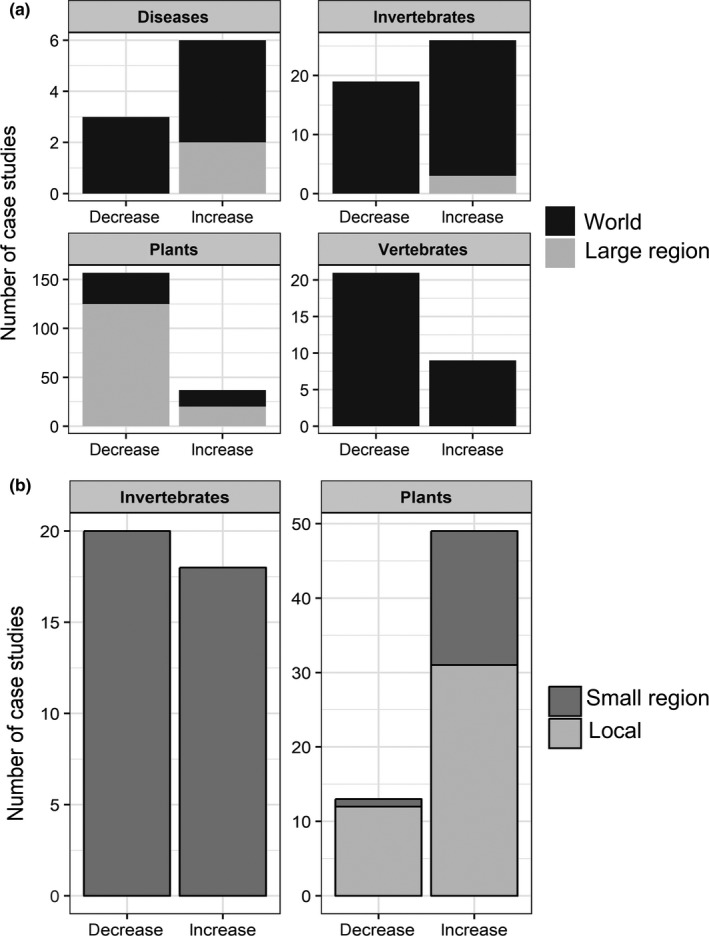
Number of case studies that predicted decrease or increase in range size following by climate change at (a) the world and large region for diseases, invertebrates, plants, and vertebrates and (b) for small region and local scales for both invertebrates and plants (See Table S2 for figures details per taxa)

More case studies predicted a decrease in IAS range following climate change (*N* = 233) than an increase (*N* = 145), while few studies predicted no change (Figure 2 and Table S2). This trend was particularly marked for studies at world and large region scales, and for studies of plants and vertebrates. Specifically, the number of case studies for plants or vertebrates that predicted a decrease in range is more than three or two times, respectively, the number of case studies predicting an increase of species range size. There was an opposite trend for invertebrates (especially arthropods and molluscs) and diseases. Indeed, a larger number of case studies predicted range expansions following climate change at regional scale (sample sizes were low for diseases, though) than contractions. At the small region scale, we found opposite results for invertebrates and plants as compared to larger scales. Here, the majority of case studies on plants predicted an increase in range following climate change, and those for invertebrates predicted a decrease.

**Figure 3:**
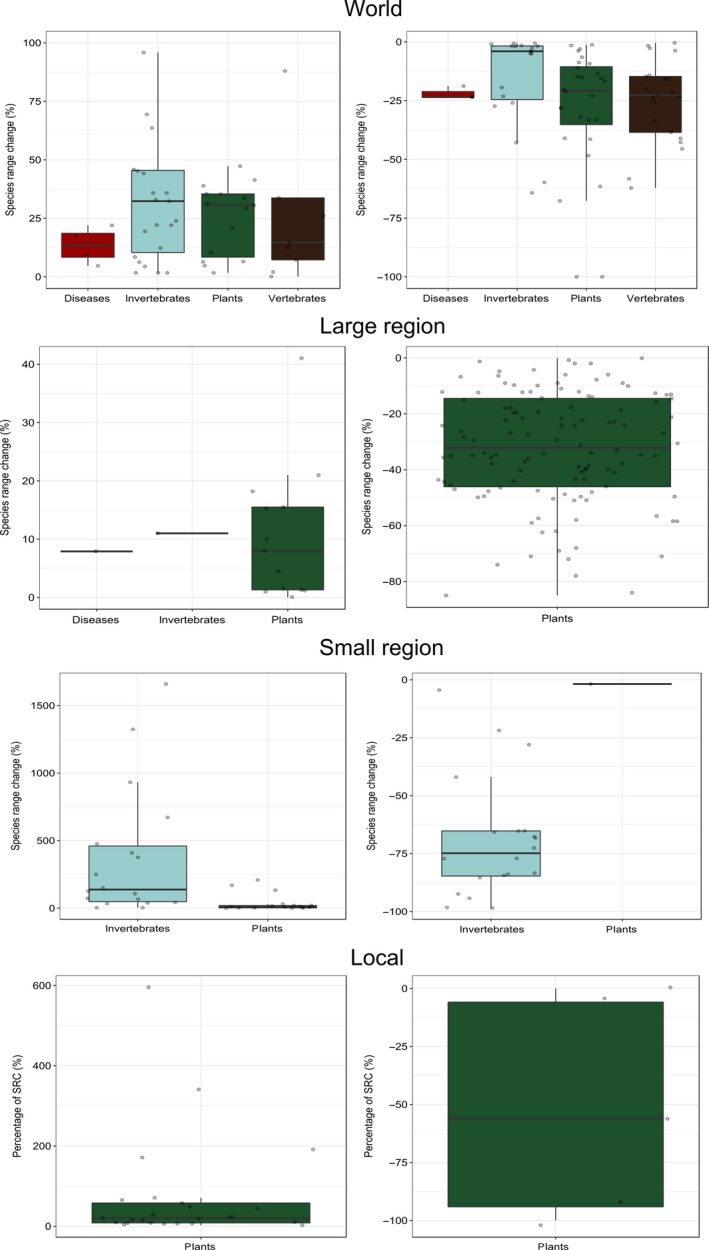
Invader range size responses in percentage at world, large and small regional scale as well as local scale for diseases, invertebrates, plants, and vertebrates

We also analyzed the SRC values that were predicted for each case study. At the world scale, we found that on average species predicted to decrease were projected to do so by −24%, whereas species predicted to increase in range were projected to do so by +35%. Median values of decrease in range were −20% and increase of +24%. Plants showed the same trend with predicted increases higher than decreases at world (+31% vs. −21%) and smaller regions (+11% vs. −2%). Regarding world‐scale studies, we also found predicted increases in range to be higher than decreases for invertebrates (+32% vs. −4%), especially for molluscs (+22% vs. −1%) (Figure 3). In contrast, the predicted decreases in range for vertebrates were higher in magnitude than the predicted increases (+15% vs. −23%), although the results for mammals (+14% vs. −16%) were similar in magnitude. Similarly, there were higher predicted decreases than increases in range for diseases (−23% vs. +13%). In contrast, for the large region, plant range sizes were predicted to decrease more than to increase (−32% vs. +8%). For the small region and local scales, we found similar results for plants and invertebrates, with a larger predicted increase in range than decrease. However, upper ranges values of invertebrates and plants at local scale could be mainly due to strong outliers.

**Figure 4:**
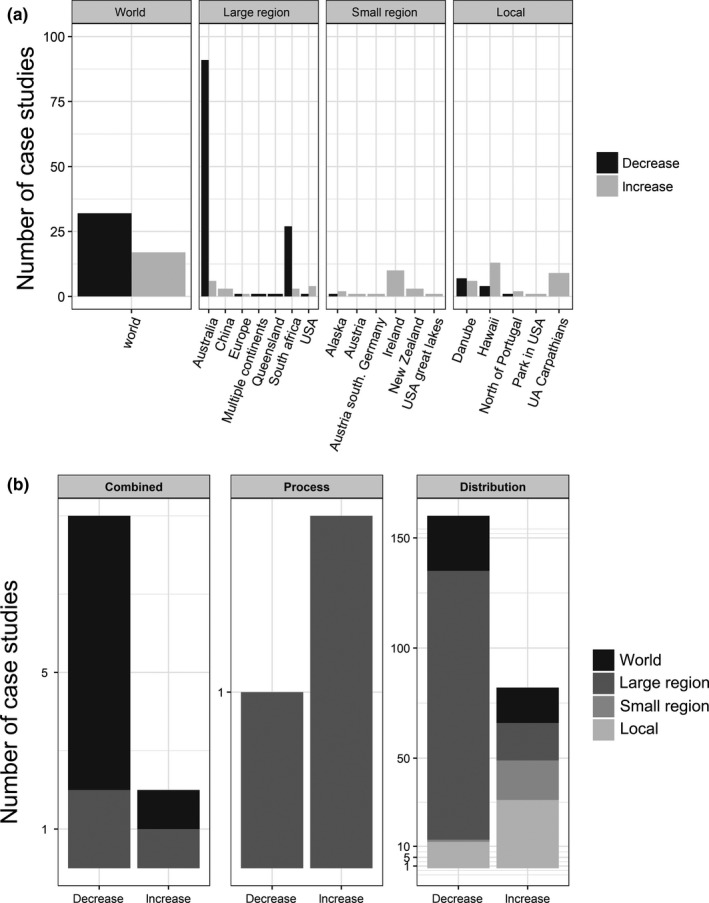
Number of case studies predicted to either decrease or increase for plants following climate change. (a) At different spatial scales, with details of increase and decrease. (b) For the three main modeling approaches: based on combined approaches (e.g., Climex), based on process only, and based on distribution only (e.g., BioMod)

Focusing on plants, we found that most plant ranges are predicted to decrease at the world and large spatial scales. This is particularly true for Australia and South Africa (Figure 4A). At smaller scales, we observed a larger number of case studies that predicted an increase in plant range size, especially in Ireland and Hawaii. We also explored if differences between studies in predicting decreases versus increases in ranges were related to approaches specific to the studies (Figure 4B). We might expect that approaches based on distribution only are more likely to overestimate the risk of invasions, as they assume species survival without considering variation in phenology or growth rate. Yet we found that results were similar across approaches, with both approaches showing a larger number of case studies predicted to decrease in range at large spatial scales. However, note that the strong bias toward the use of distribution models means that the comparison is rather weak.

## 4 DISCUSSION

Given the tremendous ecological impacts of invasive species and recent concerns over the potential effects of climate change on their distributions, we urgently need to investigate these effects. We observed an increasing number of studies that investigate this issue, especially since 2009. The small number of studies before 2009 could be surprising as the introduction of MaxEnt, BioMod, and Climex occurred before this date (Phillips, Anderson, & Schapire, 2006; Sutherst, 2003; Thuiller, 2003). Yet, the release of multimodel datasets to explore future climate scenarios in 2007 as well as MaxEnt documentation and BioMod ensemble modeling in 2009 may have contributed to increase the visibility and use on such approaches.

One of our key findings is that plant and vertebrate ranges are more frequently predicted to decrease than increase at large spatial scales. A potential explanation would be that the predicted decrease in range size is mainly due to oceans acting as barriers to species movements following climate change, especially in Europe and Australia because the predicted shifts in range due to climate change are generally poleward (Parmesan & Yohe, 2003). For instance, vertebrates studied here are bias toward mammals that are mostly problematic in Europe and Australia, where the suitable conditions are predicted to shift at higher latitudes (Bellard et al., 2013). Similarly, in Australia, predicted shifts in plants were likely truncated because of oceanic barriers (Figure 4).

On the other hand, at smaller scales where most management actions are undertaken, many case studies predicted an increase in the range of plants. For instance, in certain countries such as the USA, Ireland, and China, studies predict that plants range sizes will increase. This might be due to the fact that climate is not a limiting factor at such scales, whereas other important yet potentially limiting factors such as land use, soils, and species interactions (Bradley & Mustard, 2006; Morales‐Castilla, Matias, Gravel, & Araújo, 2015) are rarely considered in such studies. For instance, in Ireland, increases in range sizes of aquatic plants were found with global climate models, with shifts toward northern parts of the islands. Yet, when the authors also included a regional model that encompassed land use and nutrient variables, the projected ranges decreased by more than twofold (Kelly, Leach, Cameron, Maggs, & Reid, 2014).

In small island ecosystems, we might expect a decrease in predicted range sizes due to ocean barriers. Yet in Hawaii, we observed a clear increase of SRC (Vorsino et al., 2014). The authors of this study argued that given its limited latitudinal distribution, plant species need to migrate to upper elevation habitats to find temperature equivalent zones, which is the case for almost two‐thirds of the studied plants, except for plants that already occupied upper elevation wet forest habitats, which explain their predicted decrease in range sizes. In contrast, invertebrates (especially molluscs) and diseases were predicted to increase in range at large spatial scales and to decrease at smaller scales. However, given the small sample size of case studies at local scales, we recommend extreme caution in drawing conclusions at this scale. Overall, the small sample size prevents us from disentangling the scale effects from other conflated factors such as island versus mainland or taxonomic groups.

Another striking result is the contrast between the *large* number of case studies that predicted ranges to decrease and the *small* magnitude of the predicted decrease. By comparison, there were fewer case studies where ranges were predicted to increase, yet these had a *greater* magnitude (see methods for details). For example, while most case studies revealed a potential decrease in range size for plants, the magnitude of such decreases is only −21% (predicted increase +31%), revealing a worrying opportunity of known invaders currently limited by climate conditions to expand in the future. Yet, the potential increase could be overestimated because studies mostly considered climate as the only limiting factor for species distribution (without considering available resources, habitat, capacity to invade those new areas). In contrast, if the climate is not suitable, it is unlikely the species will survive except if the species is able to adapt. Hence, the extent of increase in range should be treated with more caution than a decrease.

While some conclusions could be drawn from our findings, it is important to emphasize certain gaps and biases. First, only temperature and precipitation changes were generally considered in modeling studies (Table S3). The direct effects of elevated CO_2_ itself have not been incorporated into the predictions, despite the strong influence this may have on plant's invasiveness. For instance, other studies have shown that elevated CO_2_ can increase productivity, competitiveness, and invasion success of plants relative to native species (Dukes, 2002; Smith et al., 2000). The effects of land‐use type were considered in only 134 case studies, while it is known to have a strong effect on IAS distributions (Chytrý et al., 2012) and to increase invasibility (Mattingly & Orrock, 2013). We found only one study that explicitly explored the combined effects of propagule pressure and climate change, while a few others considered it indirectly through road density (West et al., 2015) or a human influence index (Qin, Zhang, DiTommaso, Wang, & Liang, 2016).

Second, our analyses focused on species that are already known to be established, thus other alien species—those that are not (yet) established or impact ecosystems because of a time‐lag between the introduction and the invasion phases (Gassó, Pyšek, Vilà, & Williamson, 2010) or are invasive but for which this is currently unknown—were excluded.

As a result, there is clear room for improvement. Our dataset included about 350 IAS (including a large majority of well‐known invaders), but there are other IAS occurring worldwide, which should be studied as well. In addition, our review is not exhaustive (e.g., non‐English language studies, studies conducted in 2017), and the studies considered here represent only a sample of the literature on this topic. Studies that explicitly focused on particular species without mentioning higher taxa (e.g., plant, vertebrate, invertebrate, alga, bacteri, virus, microorganism fungi) within their title, keywords, or abstract were definitely to be missed by our search terms. To be inclusive, our search string should have omitted higher taxa information, and this result in more than 18,000 publications. Such a large number of studies would have been difficult to screen, and thus, we focused the analyses on a sample of the literature and did not aim to be exhaustive.

Third, most modeling studies have only addressed the establishment and spread of IAS, but invasion processes include other stages like transport and introduction that are often neglected (Blackburn et al., 2011). As there is evidence that rates of transport and introduction of IAS might increase with global change (Hellmann et al., 2008), it will be crucial to examine the effects of climate change over the entire invasion process (see, e.g., Early et al., 2016). In addition, mechanisms of invasion debt are also rarely considered or mentioned in such studies, while the timing to manage IAS might be crucial. Climate change will also affect native biodiversity, alien species resources, allelopathy, land use, and biotic interactions among species and ecosystems (Bellard, Bertelsmeier, Leadley, Thuiller, & Courchamp, 2012; Oliver & Morecroft, 2014; Walther, 2010). Thus, the frameworks provided by Blackburn and colleagues regarding both the different stages of invasions and the multitude of impacts from individuals to ecosystems should be considered in future assessments (Blackburn et al., 2011, 2014) as well as future land use and biotic interactions.

### 4.1 Management of invaders following climate change

Despite the uncertainties mentioned above, these results provide useful information for species management. It is now established that trade and transport of alien species are principal determinants of IAS distributions worldwide (Hulme, 2009; Seebens, Gastner, & Blasius, 2013), but climate change will alter those distributions in the future (Bellard et al., 2013). Our results here reaffirm the major role of climate change as a driver of IAS distribution in the future. Therefore, we need to consider these drivers jointly in order to tackle IAS issues and better inform management decisions.

Second, our results indicate that climate change may sometimes offer opportunities to more efficiently manage IAS, in agreement with Bradley et al. (2009). We should take advantage of these opportunities by both inhibiting introductions to areas where climate might become suitable, and implementing eradication programs where climate might become unsuitable. Our findings suggest there will be major changes in current distributions of IAS, which will lead to significant modifications of biodiversity patterns through decline in native population, genetic and functional diversity, as we already observed during the past decades (Simberloff et al., 2013). For instance, a decrease in range size accompanied by a shift of the species range into new areas can have devastating effects on the invaded communities. In addition, we also need to consider range shifts of native species, increasing numbers of extreme events, environmental changes, and trophic mismatches, which can make it easier for IAS to invade. A recent review showed that most native plant species will not be able to track suitable climate conditions fast enough (Corlett & Westcott, 2013); however, the movements of alien species are facilitated by humans, and thus, alien species might move faster than native species. Overall, our results imply that current IAS are unlikely to stop their spread and many will be able to invade new areas following climate change. Therefore, stringent policies and control should be implemented to mitigate future effects of invasions.

Modeling has been used to identify historical areas of species distribution (Maiorano et al., 2013), to predict sites of potential presence for rare species (Raxworthy et al., 2003), and to support conservation planning and sanctuary selection for endangered species (Araújo, Cabeza, Thuiller, Hannah, & Williams, 2004; Leroy et al., 2014). We suggest that these approaches combined with local knowledge and studies can also inform policymakers and managers about future areas at risk from IAS. For instance, predictions over a short time scale (i.e*.,* for 2020) should be used to establish lists of invaders to be banned from countries and trade zones. At the moment, such lists are mainly established through current risk posed by invaders through knowledge of past introductions, spread, and impacts (Kumschick & Richardson, 2013). The establishment of a formal procedure that includes potential risk of invasions following climate change to determine invaders of high concern for management should be prioritized. Because shifting species’ ranges respect neither political borders nor protected areas, cooperation between countries will be crucial to fight further invasions (Walther et al., 2009).

As invasion debts are likely to occur during invasion processes (Rouget et al., 2016), it is also necessary to consider not only past information but also future potential risk. The different kinds of results for plants and invertebrates between local (applied management measures) and large spatial scales (implementation of policies) suggest the need for caution. In particular, the small number of studies that focus on local as compared to large areas demands to investigate local spatial scales for areas of particular interest with approaches and techniques adapted for local‐scale studies (Pearson and Dawson 2003). Ultimately, our ability to accurately predict species distribution in the future will depend on our capacity to improve current tools and go beyond present approaches and concepts.

### 4.2 Currents gaps in our knowledge and future improvements

#### 4.2.1 Current biases

Our analyses revealed a taxonomic bias toward plants and invertebrates, while invasive vertebrates are understudied although they are mainly responsible for past known extinctions worldwide (Bellard, Cassey et al., 2016). Note that this bias could be due to our search terms that are most likely to capture studies with unknown invertebrates (in which invertebrates is cited in the title or keywords) than studies focusing on well‐known vertebrates (as the name of the species is mentioned, the term vertebrates might be useless). Our analyses also revealed that aquatic invaders are understudied in the context of climate change. This is also true for the broader context of biological invasions (Lowry et al., 2012). A significant number of case studies were conducted at the world scale. Yet, we still observed a strong geographic bias of the case studies toward Australia, Europe, South Africa, and the United States. This observation adds to previous analyses which also found geographic biases in research on biological invasions (Bellard & Jeschke, 2016; Lowry et al., 2012). As a result, most of the research on IAS and climate change is conducted on species that affect upper‐middle to high‐income countries. Yet, it is most likely that future hotspots of invasions might be located in newly industrialized and developing economies (see Dyer et al., 2017 for birds). Another potential bias comes from the lack of distinction between native and alien ranges where climate change effects can be divergent, as recently suggested for three invasive freshwater macrophytes that were predicted to increase in their alien range but decrease in their native range (Gillard, Thiebaut, Deleu, & Leroy, 2017). Although the most damaging impacts from some IAS occur on islands (Bellard, Rysman, Leroy, Claud, & Mace, 2017) and eradication programs are mainly implemented there (Jones et al., 2016), most of the studies analyzed here focused on mainland systems (except for Hawaii and New Zealand).

Invasion processes can take place over decades including transport and introduction, through establishment to spread, and all of these stages can be altered by climate change (Hellmann et al., 2008). In addition, current approaches consider a homogeneous risk of invasion in the predicted suitable areas, but it is widely acknowledged that invader impacts depend on the local context (Pyšek et al., 2012). The multiple impacts of invaders include homogenization of the biota, loss of genetic and functional diversity, and disturbance of ecosystem services, but currently available studies only focus on the distribution of invaders without assessing the impacts of future invasions. Therefore, future research should move to consider in more detail the potential impacts on native biodiversity and society.

#### 4.2.2 Limitations of modeling techniques

As the papers reviewed here are based on modeling techniques, they are subject to the general limitations of these techniques which have been extensively covered in the literature (Beaumont, Hughes, & Pitman, 2008; Senay, Worner, & Ikeda, 2013; Stoklosa, Daly, Foster, Ashcroft, & Warton, 2014; Wiens, Stralberg, Jongsomjit, Howell, & Snyder, 2009). There are additional limitations specific to the application of these techniques to IAS in the face of climate change, which we describe here. A plethora of methods exist for modeling species distributions, and none of them is specific to IAS. Among the most used approaches, BioMod and MaxEnt are purely correlative models, and Climex also relies on the species distribution but has a more process‐based orientation (Elith, 2015). Most studies in our dataset used correlative approaches rather than mechanistic models. We may expect that correlative approaches based on distribution only are more likely to overestimate the risk posed by IAS as they do not consider key processes like growth rate (reproductive strategy, phenology) that may limit the future distribution of species. Yet, we did not find differences between results from distribution only techniques and process and distribution techniques (Figure 4B), although the number of studies based on process only is low. In this study, we focus on main trends regarding the predicted effect of climate change on IAS distributions, but further analyses to investigate how modeling choices (e.g., algorithm, resolution) may influenced those results are also needed. Therefore, the predicted species range changes is an estimation and do not exactly reflect the future realized distribution of the species. This would require also considering biotic interactions and potential resources.

##### Equilibrium hypothesis

Correlative distribution models assume that species niches do not change over time or space. This assumption is known to be violated by IAS which have been shown to occupy different niches in their invaded range (Broennimann et al., 2007; but see also Petitpierre et al., 2012) or to shift their realized niche (Escobar, Qiao, Phelps, Wagner, & Larkin, 2016) because of altered biotic interactions (e.g., loss of major competitors) or evolutionary changes (Jeschke & Strayer, 2008; Lavergne, Mouquet, Thuiller, & Ronce, 2010; Moran & Alexander, 2014). This challenge is even more problematic in the case of predicting future IAS distributions, as climate change may create novel conditions (Williams, Jackson, & Kutzbach, 2007) (see below) and lead to novel biotic interactions which have not been encountered by the species in their eco‐evolutionary history (Saul & Jeschke, 2015). Correlative distribution models alone may be insufficient to overcome this challenge, and more complex models including biotic interactions are necessary (Blois, Zarnetske, Fitzpatrick, & Finnegan, 2013; Jeschke & Strayer, 2008). A promising way forward to reach this objective will be to combine propagule pressure analysis, correlative approaches (Fletcher, Gillingham, Britton, Blanchet, & Gozlan, 2016), and modeling species co‐occurrences of associated species (Morelli & Tryjanowski, 2015; or to constrain the composition of species assemblages, Guisan & Rahbek, 2011) to provide inference about likely interspecific effects in ecosystems.

##### Novel climate combinations

An additional shortcoming of many current studies is that predictions are made beyond the domain of parameter calibration. The inclusion of data from both the native and invaded range has been recommended as a necessary step to overcome this challenge (Broennimann & Guisan, 2008). The application of process‐based models such as Climex is useful here, as they have better extrapolation potential than correlative models (Dormann et al., 2012). However, their data requirements and assumptions may limit their applicability (Box 1). Moreover, correlative approaches are unable to deal with new combinations of climate that will mainly occur at low latitudes under climate change (Williams et al., 2007; Bellard et al., 2014, but Climex seems more appropriate to deal with these novel climates. Overall, mapping novel environments is important to assess robustness of model outputs and guide users as to where predictions may be highly unreliable (Elith, 2015). For example, in the BioMod R platform, a “clamping mask” function allows users to map projections beyond the domain of parameter calibration.

##### Model evaluation

A further challenge comes from the evaluation of model predictive performance. Current robustness of approaches is evaluated through metrics like the area under the receiver operating characteristic (known as AUC) or the true skill statistic (known as TSS) that have been highly criticized (Allouche, Tsoar, & Kadmon, 2006; Leroy et al., 2017; Lobo, Jiménez‐Valverde, & Real, 2008; Somodi et al. 2017). More attention should be given to the problem of evaluation, as this is a fundamental way to communicate about uncertainty. Specifically, in the case of biological invasions, we want to know whether models tend to over‐ or underestimate the risk posed by invaders (Márcia Barbosa, Real, Muñoz, & Brown, 2013). A potential solution lies in evaluating models with respect to biological knowledge (Mainali et al., 2015), interpret within the limits of observation data (Guillera‐Arroita et al., 2015; Lahoz‐Monfort, Guillera‐Arroita, & Wintle, 2013) and apply alternative metrics (Márcia Barbosa et al., 2013). Developing such metrics will be highly valuable for managers and policymakers to establish prioritization of invaders and implement eradication programs. Another way to assess our capacity to predict invasion risk is to parameterize models based on historic data: predictions of such models can be compared to current actual ranges, hence giving an estimate of a model's accuracy (Jeschke & Strayer, 2008).

Despite current limitations, modeling studies have been widely applied to guide management decisions (Giljohann, Hauser, Williams, & Moore, 2011) and have recently been shown to reflect the correct trend when predicting climate change impacts (Stephens et al., 2016). Taking into account, the considerations above and moving forward to improve current practice should be a priority.

## CONFLICT OF INTEREST

The authors have no conflict of interest to declare.

## AUTHOR CONTRIBUTIONS

C.B. collected the data, did the analyses, and wrote the first draft of the article. J.M.J., B.L., and G.M.M. all contributed to the writing and interpreting the results.
