# Supplementary material for: Insights from modeling studies on how climate change affects invasive alien species geography
Source: Ecol Evol. 2018 May 4;8(11):5688–700. doi: 10.1002/ece3.4098 (PMC6010883; doi:10.1002/ece3.4098)
Supplement: Supplementary file 1 [file ECE3-8-5688-s001.docx]

**Insights from modelling studies on how climate change affects invasive alien species geography**

C. Bellard, J. M. Jeschke, B. Leroy & G. M. Mace

**Table S1:** Publications used in the current study N=71

| References | Year | Title |
| --- | --- | --- |
| Aurambout, J. P.; Finlay, K. J.; Luck, J.; Beattie, G. A. C. | 2009 | A concept model to estimate the potential distribution of the Asiatic citrus psyllid (Diaphorina citri Kuwayama) in Australia under climate change-A means for assessing biosecurity risk |
| Bacon. Steven J.; Aebi. Alexandre; Calanca. Pierluigi; Bacher. Sven | 2014 | Quarantine arthropod invasions in Europe: the role of climate. hosts and propagule pressure |
| Barbet-Massin, Morgane; Rome, Quentin; Muller, Franck; Perrard, Adrien; Villemant, Claire; Jiguet, Frederic | 2013 | Climate change increases the risk of invasion by the Yellow-legged hornet |
| Beaumont. Linda J.; Gallagher. Rachael V.; Downey. Paul O.; Thuiller. Wilfried; Leishman. Michelle R.; Hughes. Lesley | 2009 | Modelling the impact of Hieracium spp. on protected areas in Australia under future climates |
| Beaumont. Linda J.; Gallagher. Rachael V.; Leishman. Michelle R.; Hughes. Lesley; Downey. Paul O. | 2014 | How can knowledge of the climate niche inform the weed risk assessment process? A case study of Chrysanthemoides monilifera in Australia |
| Beaumont. Linda J.; Gallagher. Rachael V.; Thuiller. Wilfried; Downey. Paul O.; Leishman. Michelle R.; Hughes. Lesley | 2009 | Different climatic envelopes among invasive populations may lead to underestimations of current and future biological invasions |
| Bellard, Celine; Thuiller, Wilfried; Leroy, Boris; Genovesi, Piero; Bakkenes, Michel; Courchamp, Franck | 2013 | Will climate change promote future invasions? |
| Bertelsmeier, Cleo; Luque, Gloria M.; Courchamp, Franck | 2013 | Increase in Quantity and Quality of Suitable Areas for Invasive Species as Climate Changes |
| Bertelsmeier, Cleo; Luque, Gloria M.; Courchamp, Franck | 2013 | Global warming may freeze the invasion of big-headed ants |
| Bertelsmeier, Cleo; Luque, Gloria M.; Hoffmann, Benjamin D.; Courchamp, Franck | 2015 | Worldwide ant invasions under climate change |
| Bourdot, Graeme W.; Lamoureaux, Shona L.; Watt, Michael S.; Manning, Lucy K.; Kriticos, Darren J. | 2012 | The potential global distribution of the invasive weed Nassella neesiana under current and future climates |
| Bradley, Bethany A. | 2009 | Regional analysis of the impacts of climate change on cheatgrass invasion shows potential risk and opportunity |
| Bradley. Bethany A.; Oppenheimer. Michael; Wilcove. David S. | 2009 | Climate change and plant invasions: restoration opportunities ahead? |
| Broennimann. Olivier; Guisan. Antoine | 2008 | Predicting current and future biological invasions: both native and invaded ranges matter |
| Chejara, V. K.; Kriticos, D. J.; Kristiansen, P.; Sindel, B. M.; Whalley, R. D. B.; Nadolny, C. | 2010 | The current and future potential geographical distribution of Hyparrhenia hirta |
| de Rivera, Catherine E.; Steves, Brian P.; Fofonoff, Paul W.; Hines, Anson H.; Ruiz, Gregory M. | 2011 | Potential for high-latitude marine invasions along western North America |
| Domisch, Sami; Jaehnig, Sonja C.; Haase, Peter | 2011 | Climate-change winners and losers: stream macroinvertebrates of a submontane region in Central Europe |
| Dzigurski, D.; Ljevnaic-Masic, B.; Milic, D.; Acanski, J. | 2015 | Impact of climate changes on aquatic vegetation of hydromeliorative facilities |
| Fand, Babasaheb B.; Tonnang, Henri E. Z.; Kumar, Mahesh; Bal, Santanu K.; Singh, Naveen P.; Rao, D. V. K. N.; Kamble, Ankush L.; Nangare, Dhananjay D.; Minhas, Paramjit S. | 2014 | Predicting the impact of climate change on regional and seasonal abundance of the mealybug Phenacoccus solenopsis Tinsley (Hemiptera: Pseudococcidae) using temperature-driven phenology model linked to GIS |
| Fennell, Mark; Murphy, James E.; Gallagher, Tommy; Osborne, Bruce | 2013 | Simulating the effects of climate change on the distribution of an invasive plant, using a high resolution, local scale, mechanistic approach: challenges and insights |
| Fischer. Dominik; Thomas. Stephanie M; Suk. Jonathan E; Sudre. Bertrand; Hess. Andrea; Tjaden. Nils B; Beierkuhnlein. Carl; Semenza. Jan C | 2013 | Climate change effects on Chikungunya transmission in Europe: geospatial analysis of vector's climatic suitability and virus' temperature requirements. |
| Gallagher, R. V.; Duursma, D. Englert; O'Donnell, J.; Wilson, P. D.; Downey, P. O.; Hughes, L.; Leishman, M. R. | 2013 | The grass may not always be greener: projected reductions in climatic suitability for exotic grasses under future climates in Australia |
| Gallagher, Rachael V.; Hughes, Lesley; Leishman, Michelle R.; Wilson, Peter D. | 2010 | Predicted impact of exotic vines on an endangered ecological community under future climate change |
| Ganley, Rebecca J.; Watt, Michael S.; Kriticos, Darren J.; Hopkins, Anna J. M.; Manning, Lucy K. | 2011 | Increased risk of pitch canker to Australasia under climate change |
| Gilioli. Gianni; Pasquali. Sara; Parisi. Simone; Winter. Stephan | 2014 | Modelling the potential distribution of Bemisia tabaci in Europe in light of the climate change scenario |
| Gonzalez-Munoz, N.; Linares, J. C.; Castro-Diez, P.; Sass-Klaassen, U. | 2014 | Predicting climate change impacts on native and invasive tree species using radial growth and twenty-first century climate scenarios |
| Gonzalez-Munoz, Noelia; Bellard, Celine; Leclerc, Camille; Meyer, Jean-Yves; Courchamp, Franck | 2015 | Assessing current and future risks of invasion by the \green cancer\" Miconia calvescens" |
| Hager, Heather A.; Sinasac, Sarah E.; Gedalof, Ze'ev; Newman, Jonathan A. | 2014 | Predicting Potential Global Distributions of Two Miscanthus Grasses: Implications for Horticulture, Biofuel Production, and Biological Invasions |
| Harrigan, Ryan J.; Thomassen, Henri A.; Buermann, Wolfgang; Smith, Thomas B. | 2014 | A continental risk assessment of West Nile virus under climate change |
| Hoveka, L. N.; Bezeng, B. S.; Yessoufou, K.; Boatwright, J. S.; Van der Bank, M. | 2016 | Effects of climate change on the future distributions of the top five freshwater invasive plants in South Africa |
| Ibanez, Ines; Silander, John. A., Jr.; Allen, Jenica M.; Treanor, Sarah A.; Wilson, Adam | 2009 | Identifying hotspots for plant invasions and forecasting focal points of further spread |
| Jara, Valentina; Meza, Francisco J.; Zaviezo, Tania; Chorbadjian, Rodrigo | 2013 | Climate change impacts on invasive potential of pink hibiscus mealybug, Maconellicoccus hirsutus (Green), in Chile |
| Jarnevich, Catherine S.; Holcombe, Tracy R.; Bella, Elizabeth M.; Carlson, Matthew L.; Graziano, Gino; Lamb, Melinda; Seefeldt, Steven S.; Morisette, Jeffery | 2014 | Cross-Scale Assessment of Potential Habitat Shifts in a Rapidly Changing Climate |
| Jones, Miranda C.; Dye, Stephen R.; Pinnegar, John K.; Warren, Rachel; Cheung, William W. L. | 2013 | Applying distribution model projections for an uncertain future: the case of the Pacific oyster in UK waters |
| Kelly, Ruth; Leach, Katie; Cameron, Alison; Maggs, Christine A.; Reid, Neil | 2014 | Combining global climate and regional landscape models to improve prediction of invasion risk |
| Kleinbauer, I.; Dullinger, S.; Peterseil, J.; Essl, F. | 2010 | Climate change might drive the invasive tree Robinia pseudacacia into nature reserves and endangered habitats |
| Koncki, Natalie G.; Aronson, Myla F. J. | 2015 | Invasion Risk in a Warmer World: Modeling Range Expansion and Habitat Preferences of Three Nonnative Aquatic Invasive Plants |
| Kriticos, D. J.; Sutherst, R. W.; Brown, J. R.; Adkins, S. W.; Maywald, G. F. | 2003 | Climate change and biotic invasions: a case history of a tropical woody vine |
| Kriticos, D. J.; Watt, M. S.; Potter, K. J. B.; Manning, L. K.; Alexander, N. S.; Tallent-Halsell, N. | 2011 | Managing invasive weeds under climate change: considering the current and potential future distribution of Buddleja davidii |
| Kriticos, DJ; Sutherst, RW; Brown, JR; Adkins, SW; Maywald, GF | 2003 | Climate change and the potential distribution of an invasive alien plant: Acacia nilotica ssp indica in Australia |
| Kroschel, J.; Sporleder, M.; Tonnang, H. E. Z.; Juarez, H.; Carhuapoma, P.; Gonzales, J. C.; Simon, R. | 2013 | Predicting climate-change-caused changes in global temperature on potato tuber moth Phthorimaea operculella (Zeller) distribution and abundance using phenology modeling and GIS mapping |
| Mazur, Martha L. Carlson; Kowalski, Kurt P.; Galbraith, David | 2014 | Assessment of suitable habitat for Phragmites australis (common reed) in the Great Lakes coastal zone |
| Michael, Pippa J.; Yeoh, Paul B.; Scott, John K. | 2012 | The current and future projected distribution of Solanum hoplopetalum (Solanaceae): an indigenous weed of the south-western Australian grain belt |
| Michael, Pippa J.; Yeoh, Paul B.; Scott, John K. | 2012 | Potential Distribution of the Australian Native Chloris truncata Based on Modelling Both the Successful and Failed Global Introductions |
| Murray, Justine V.; Stokes, Kate E.; van Klinken, Rieks D. | 2012 | Predicting the potential distribution of a riparian invasive plant: the effects of changing climate, flood regimes and land-use patterns |
| O'Donnell, Jessica; Gallagher, Rachael V.; Wilson, Peter D.; Downey, Paul O.; Hughes, Lesley; Leishman, Michelle R. | 2012 | Invasion hotspots for non-native plants in Australia under current and future climates |
| Padalia, Hitendra; Srivastava, Vivek; Kushwaha, S. P. S. | 2015 | How climate change might influence the potential distribution of weed, bushmint (Hyptis suaveolens)? |
| Parker-Allie, F.; Musil, C. F.; Thuiller, W. | 2009 | Effects of climate warming on the distributions of invasive Eurasian annual grasses: a South African perspective |
| Patz, JA; Martens, WJM; Focks, DA; Jetten, TH | 1998 | Dengue fever epidemic potential as projected by general circulation models of global climate change |
| Porter. Jh; parry. Ml; carter. Tr | 1991 | The potential effects of climatic-change on agricultural insect pests |
| Qin, Z.; DiTommaso, A.; Wu, R. S.; Huang, H. Y. | 2014 | Potential distribution of two Ambrosia species in China under projected climate change |
| Qin, Z.; Zhang, J. E.; DiTommaso, A.; Wang, R. L.; Liang, K. M. | 2016 | Predicting the potential distribution of Lantana camara L. under RCP scenarios using ISI-MIP models |
| Richardson, David M.; Iponga, Donald M.; Roura-Pascual, Nuria; Krug, Rainer M.; Milton, Suzanne J.; Hughes, Gregory O.; Thuiller, Wilfried | 2010 | Accommodating scenarios of climate change and management in modelling the distribution of the invasive tree Schinus molle in South Africa |
| Richter, Robert; Berger, Uwe E.; Dullinger, Stefan; Essl, Franz; Leitner, Michael; Smith, Matthew; Vogl, Gero | 2013 | Spread of invasive ragweed: climate change, management and how to reduce allergy costs |
| Shabani, Farzin; Kumar, Lalit | 2013 | Risk Levels of Invasive Fusarium oxysporum f. sp in Areas Suitable for Date Palm (Phoenix dactylifera) Cultivation under Various Climate Change Projections |
| Sheppard, Christine S. | 2013 | Potential spread of recently naturalised plants in New Zealand under climate change |
| Simpson, Maria; Prots, Bohdan | 2013 | Predicting the distribution of invasive plants in the Ukrainian Carpathians under climatic change and intensification of anthropogenic disturbances: implications for biodiversity conservation |
| Sims-Chilton, N. M.; Zalucki, M. P.; Buckley, Y. M. | 2010 | Long term climate effects are confounded with the biological control programme against the invasive weed Baccharis halimifolia in Australia |
| Storkey. Jonathan; Stratonovitch. Pierre; Chapman. Daniel S.; Vidotto. Francesco; Semenov. Mikhail A. | 2014 | A Process-Based Approach to Predicting the Effect of Climate Change on the Distribution of an Invasive Allergenic Plant in Europe |
| Sykes, MT | 2001 | Modelling the potential distribution and community dynamics of lodgepole pine (Pinus contorta Dougl. ex. Loud.) in Scandinavia |
| Taylor, Subhashni; Kumar, Lalit | 2013 | Potential distribution of an invasive species under climate change scenarios using CLIMEX and soil drainage: A case study of Lantana camara L. in Queensland, Australia |
| Taylor, Subhashni; Kumar, Lalit; Reid, Nick; Kriticos, Darren J. | 2012 | Climate Change and the Potential Distribution of an Invasive Shrub, Lantana camara L. |
| van Klinken, Rieks D.; Lawson, Ben E.; Zalucki, Myron P. | 2009 | Predicting invasions in Australia by a Neotropical shrub under climate change: the challenge of novel climates and parameter estimation |
| Vicente, J. R.; Fernandes, R. F.; Randin, C. F.; Broennimann, O.; Goncalves, J.; Marcos, B.; Pocas, I.; Alves, P.; Guisan, A.; Honrado, J. P. | 2013 | Will climate change drive alien invasive plants into areas of high protection value? An improved model-based regional assessment to prioritise the management of invasions |
| Vorsino, Adam E.; Fortini, Lucas B.; Amidon, Fred A.; Miller, Stephen E.; Jacobi, James D.; Price, Jonathan P.; Gon, Sam 'Ohukani'ohi'a, III; Koob, Gregory A. | 2014 | Modeling Hawaiian Ecosystem Degradation due to Invasive Plants under Current and Future Climates |
| Watt, M. S.; Kriticos, D. J.; Manning, L. K. | 2009 | The current and future potential distribution of Melaleuca quinquenervia |
| Watt, Michael S.; Kriticos, Darren J.; Lamoureaux, Shona L.; Bourdot, Graeme W. | 2011 | Climate Change and the Potential Global Distribution of Serrated Tussock (Nassella trichotoma) |
| West, Amanda M.; Kumar, Sunil; Wakie, Tewodros; Brown, Cynthia S.; Stohlgren, Thomas J.; Laituri, Melinda; Bromberg, Jim | 2015 | Using High-Resolution Future Climate Scenarios to Forecast Bromus tectorum Invasion in Rocky Mountain National Park |
| Xu, Zhonglin | 2015 | Potential distribution of invasive alien species in the upper Ili river basin: determination and mechanism of bioclimatic variables under climate change |
| Xu, Zhonglin; Feng, Zhaodong; Yang, Jianjun; Zheng, Jianghua; Zhang, Fang | 2013 | Nowhere to Invade: Rumex crispus and Typha latifolia Projected to Disappear under Future Climate Scenarios |
| Xu, Zhonglin; Peng, Huanhua; Feng, Zhaodong; Abdulsalih, Nurbay | 2014 | Predicting current and future invasion of solidago canadensis: a study from china |

**Table S2:** Invader range-size responses classified as decrease or increase at a global, regional and local scales for all groups

| Taxa | Scale | Trends | Frequency |
| --- | --- | --- | --- |
| Disease | region | increase | 2 |
| Disease | world | decrease | 3 |
| Disease | world | increase | 4 |
| Invertebrate | region | increase | 3 |
| Invertebrate | region | decrease | 20 |
| Invertebrate | region | increase | 18 |
| Invertebrate | world | decrease | 19 |
| Invertebrate | world | increase | 23 |
| Plants | local | decrease | 12 |
| Plants | local | increase | 31 |
| Plants | region | decrease | 125 |
| Plants | region | increase | 20 |
| Plants | small region | decrease | 1 |
| Plants | small region | increase | 18 |
| Plants | world | decrease | 32 |
| Plants | world | increase | 17 |
| Vertebrates | world | decrease | 21 |
| Vertebrates | world | increase | 9 |

**Table S3:** Type of variable included for each case studied.

| Variables included | Nb. Case studies |
| --- | --- |
| temperature | 423 |
| precipitation | 418 |
| land use | 134 |
| elevation | 7 |
| other variables | 158 |
